# Army Medical Corps Examinations

**Published:** 1905-03

**Authors:** 


### Army Medical Corps Examinations.

Preliminary examinations for appointment of assistant surgeons in the army will be held on May 1 and August 1, 1905, at points to be hereafter designated.

Permission to appear for examination can be obtained upon application to the surgeon general, U. S. army, Washington, D. C., from whom full information concerning the examination can be procured. The essential requirements to securing an invitation are that the applicant shall be a citizen of the United States, shall be between 21 and 31 years of age, a graduate of a medical school legally authorized to confer the degree of doctor of medicine, shall be of good moral character and habits, and shall have had at least one year's hospital training or its equivalent in practice. The examinations will be held concurrently throughout the country at points where boards can be convened. Due consideration will be given to the localities from which applications are received, in order to lessen the traveling expenses of applicants as much as possible.

In order to perfect all necessary arrangements for the examinations of May 1st, applications must be complete and in possession of the surgeon general on or before April 1st, and for the examination of August 1st, on or before July 1st. Early attention is therefore enjoined upon all intended applicants.

There are at present twenty vacancies in the medical corps of the army.

